# Supplementary material for: Immunogenicity and reactogenicity of SARS-CoV-2 vaccines BNT162b2 and CoronaVac in healthy adolescents
Source: Nat Commun. 2022 Jun 28;13:3700. doi: 10.1038/s41467-022-31485-z (PMC9240007; doi:10.1038/s41467-022-31485-z)
Supplement: Supplementary file 3 — Reporting Summary [file 41467_2022_31485_MOESM3_ESM.pdf]

nature research

Corresponding author(s): Yu Lung Lau, Wenwei Tu, Malik Peiris, Sophie Valkenburg

Last updated by author(s): May 23, 2022

Reporting Summary

Nature Research wishes to improve the reproducibility of the work that we publish. This form provides structure for consistency and transparency in reporting. For further information on Nature Research policies, see our Editorial Policies and the Editorial Policy Checklist.

Statistics

For all statistical analyses, confirm that the following items are present in the figure legend, table legend, main text, or Methods section.

n/a	Confirmed	
☐	☒	The exact sample size (*n*) for each experimental group/condition, given as a discrete number and unit of measurement
☒	☐	A statement on whether measurements were taken from distinct samples or whether the same sample was measured repeatedly
☐	☒	The statistical test(s) used AND whether they are one- or two-sided<br>*Only common tests should be described solely by name; describe more complex techniques in the Methods section.*
☐	☒	A description of all covariates tested
☐	☒	A description of any assumptions or corrections, such as tests of normality and adjustment for multiple comparisons
☐	☒	A full description of the statistical parameters including central tendency (e.g. means) or other basic estimates (e.g. regression coefficient) AND variation (e.g. standard deviation) or associated estimates of uncertainty (e.g. confidence intervals)
☐	☒	For null hypothesis testing, the test statistic (e.g. *F*, *t*, *r*) with confidence intervals, effect sizes, degrees of freedom and *P* value noted<br>*Give P values as exact values whenever suitable.*
☒	☐	For Bayesian analysis, information on the choice of priors and Markov chain Monte Carlo settings
☒	☐	For hierarchical and complex designs, identification of the appropriate level for tests and full reporting of outcomes
☐	☒	Estimates of effect sizes (e.g. Cohen's *d*, Pearson's *r*), indicating how they were calculated

Our web collection on statistics for biologists contains articles on many of the points above.

Software and code

Policy information about availability of computer code

Data collection	BD FACSDiva (version 8) and FlowJo (version 10) (Treestar) were used to collect T cell response data. WebPlotDigitizer (https://automeris.io/WebPlotDigitizer/, version 4.5) was used to extract protective efficacy plot in Khoury et al (2021, Nature Medicine) for vaccine efficacy estimation.
Data analysis	GraphPad Prism (version 9.3.1) was used to analyze immunogenicity and reactogenicity data. Power analyses were performed using G*Power (version 3.1.9.7) (Heinrich-Heine-Universität Düsseldorf, Düsseldorf, Germany) and Sampsize (version 0.6) (sampsize.sourceforge.net).

For manuscripts utilizing custom algorithms or software that are central to the research but not yet described in published literature, software must be made available to editors and reviewers. We strongly encourage code deposition in a community repository (e.g. GitHub). See the Nature Research guidelines for submitting code & software for further information.

Data

Policy information about availability of data

All manuscripts must include a data availability statement. This statement should provide the following information, where applicable:

- Accession codes, unique identifiers, or web links for publicly available datasets
- A list of figures that have associated raw data
- A description of any restrictions on data availability

The study's Protocol and Statistical Analysis Plan are contained in the Supplementary Information. To protect the confidentiality of participants, only deidentified participant-level datasets will be shared to researchers who provide a scientifically valid proposal. Since this study is ongoing, data will be available upon request 1 month after the completion of the study (anticipated in 2025). Enquiries can be addressed to lauylung@hku.hk.

Field-specific reporting

Please select the one below that is the best fit for your research. If you are not sure, read the appropriate sections before making your selection.

- [x] Life sciences
- [ ] Behavioural & social sciences
- [ ] Ecological, evolutionary & environmental sciences

For a reference copy of the document with all sections, see nature.com/documents/nr-reporting-summary-flat.pdf

Life sciences study design

All studies must disclose on these points even when the disclosure is negative.

Sample size	For primary immunogenicity objectives, when comparing the peak geometric mean (GM) immunogenicity outcomes of children with that of parents, or between vaccine types, a sample size of 61 in each group would assure that a two-sided test with a=0.05 has 99% power to detect an effect size with a Cohen's d value=0.78, or a difference of 0.51 after natural log transformation, between 2 groups and a standard deviation (SD) of 0.65 on the natural log scale within each group. For assays with higher technical requirements such as PRNT, 66 evaluable adolescents and 16 evaluable adults tested would achieve 80% power to detect the same difference with the same a and SD. For the proportion of participants with a positive result in immunogenicity outcomes or ARs, 110 adolescents would yield a 95% chance to detect the true value within +/-7.5% of the measured percentage, assuming a prevalence of 80%. Recruitment of 120 adolescents were targeted per vaccine type to accommodate for attrition.
Data exclusions	The evaluable analysis population included participants who were uninfected during the first 3 study visits (based on clinical history, baseline S-RBD IgG negativity, N and ORF8 IgG negativity), generally healthy with no major protocol deviations, blood sampling within the evaluable window for post-dose 1 (no more than 3 days earlier or later than day 21 for BNT162b2 or day 28 for CoronaVac, and before dose 2) or post-dose 2 time-points (within day 14-42 post-dose 2 and before any further doses), and had a valid result for the relevant analysis and timepoint (see protocol in Supplementary Information). The expanded analysis population included similar criteria as the evaluable population except the notable differences of the requirement of a valid immunogenicity result for the particular analysis at least 14 days post-dose 1 but before dose 2 and between 7-56 days post-dose 2 (see protocol in Supplementary Information).
Replication	Biological replicates was measured.
Randomization	This is not a randomized trial. Randomization was not planned to facilitate recruitment.
Blinding	There was no blinding in this study as randomization was not performed.

Reporting for specific materials, systems and methods

We require information from authors about some types of materials, experimental systems and methods used in many studies. Here, indicate whether each material, system or method listed is relevant to your study. If you are not sure if a list item applies to your research, read the appropriate section before selecting a response.

Materials & experimental systems

n/a	Involved in the study	
[ ]	[x]	Antibodies
[x]	[ ]	Eukaryotic cell lines
[x]	[ ]	Palaeontology and archaeology
[x]	[ ]	Animals and other organisms
[ ]	[x]	Human research participants
[ ]	[x]	Clinical data
[x]	[ ]	Dual use research of concern

Methods

n/a	Involved in the study	
[x]	[ ]	ChIP-seq
[ ]	[x]	Flow cytometry
[x]	[ ]	MRI-based neuroimaging

Antibodies

Antibodies used

S-RBD IgG ELISA:
anti-human IgG secondary antibody, Thermo Fisher Scientific, Cat# 31410, 1:5000

N and N-CTD IgG ELISA:
anti-human IgG secondary antibody, Thermo Fisher Scientific, Cat# 31410, 1:2500

S IgG ELISA:
IgG-HRP, BD, G8-185, Cat# 555788, 1:5000

Flow cytometry:
CD3, APC/Cy7, HIT3a, Biolegend, Cat# 300318, 1:60

	CD4, Pacific Blue, OKT4, Biolegend, Cat# 317429, 1:60<br>CD8, PerCP Cy5.5, HIT8a, Biolegend, Cat# 300924, 1:60<br>IFN-γ, PE, B27, Biolegend, Cat# 506507, 1:15<br>IL-2, FITC, MQ1-17H12, Biolegend, Cat# 500304, 1:15<br><br>Fixable Viability Dye, eFluor™ 506, eBioscience, Cat# 65-0866-14, 1:60<br><br>Purified anti-human CD28 Antibody, CD28.2, Biolegend, Cat# 302902, 1 µg/ml<br>Purified anti-human CD49d Antibody , 9F10, Biolegend, Cat# 304302, 1 µg/ml
Validation	Antibodies were validated by manufacturer and used as per manufacturers' instructions and previously published literature.

Human research participants

Policy information about studies involving human research participants

Population characteristics	There were 239 adolescent (11-17 years old, mean=14.0, SD=1.7) and 288 adult (18-67 years old, mean=47.5, SD=7.5) participants (total N=527), with similar numbers who completed the 2-dose (BB for BNT162b2 and CC for CoronaVac) vaccination series. Demographic characteristics were evenly distributed (Extended Data Table 1).
Recruitment	Potential participants were recruited via schools, mass media or referral in Hong Kong. Self-selection bias was deemed unlikely to influence immunogenicity outcomes in our study.
Ethics oversight	The research protocol and procedures were approved by the University of Hong Kong (HKU)/HK West Cluster Hospital Authority Institutional Review Board (UW21-157).

Note that full information on the approval of the study protocol must also be provided in the manuscript.

Clinical data

Policy information about clinical studies

All manuscripts should comply with the ICMJEguidelines for publication of clinical research and a completedCONSORT checklist must be included with all submissions.

Clinical trial registration	clinicaltrials.gov NCT04800133
Study protocol	Refer to Supplementary Information.
Data collection	Participants were enrolled between 27 April 2021 and 23 October 2021 in 2 community vaccination centers in Hong Kong.
Outcomes	The primary immunogenicity outcomes included: S-specific antibody markers, which were the S IgG and S-RBD IgG levels, sVNT % inhibition, 90% and 50% PRNT titres, S IgG avidity and FcRIIIa-binding; S-specific (and N- and M-specific for CC) IFN-+ and IL-2+ CD4+ and CD8+ T cell responses measured by the flow-cytometry-based intracellular cytokine staining assay; at 21 days post-dose 1 (or 28 days for CC) and 28 days after 2 doses at a prime-boost interval of 21 days (for BB) or 28 (for CC). The primary reactogenicity outcomes were solicited ARs and anti-pyretic use for 7 days after each vaccine dose.<br>Secondary immunogenicity outcomes included N and N-CTD IgG levels in CC recipients. For safety, the secondary outcomes were unsolicited AEs reported 28 days after each dose and SAEs collected throughout the study period. Other secondary outcomes not included in this interim analysis, such as the evaluation of similar outcomes in participants with severe paediatric illnesses, can be found in the Protocol and Statistical Analysis Plan (Supplementary Materials).

Flow Cytometry

Plots

Confirm that:

- [x] The axis labels state the marker and fluorochrome used (e.g. CD4-FITC).
- [x] The axis scales are clearly visible. Include numbers along axes only for bottom left plot of group (a 'group' is an analysis of identical markers).
- [x] All plots are contour plots with outliers or pseudocolor plots.
- [x] A numerical value for number of cells or percentage (with statistics) is provided.

Methodology

Sample preparation	Human PBMCs were isolated from whole blood using Ficoll and cryopreserved at -180 ℃ before use. Then, the PBMCs were thawed and rested for detection of SARS-Cov-2 specific T cells as described in the Online Methods.
Instrument	LSR II flow cytometer (BD, Germany)
Software	BD FACSDiva v8.0, FlowJo_V10 (Treestar)

Cell population abundance	Sorting was not performed.
Gating strategy	Lymphocytes were gated using FSC/SSC. Singlets were then selected. Viable T cells were selected upon fixable viability dye and CD3. T cells expressing CD4 or CD8 were analyzed separately for the levels of indicated cytokines.

☒ Tick this box to confirm that a figure exemplifying the gating strategy is provided in the Supplementary Information.
